# Whole Mitochondrial Genome Sequencing and Re-Examination of a Cytoplasmic Male Sterility-Associated Gene in Boro-Taichung-Type Cytoplasmic Male Sterile Rice

**DOI:** 10.1371/journal.pone.0159379

**Published:** 2016-07-14

**Authors:** Tomohiko Kazama, Kinya Toriyama

**Affiliations:** Graduate School of Agricultural Science, Tohoku University, Sendai, Japan; University of Tsukuba, JAPAN

## Abstract

Nuclear genome substitutions between subspecies can lead to cytoplasmic male sterility (CMS) through incompatibility between nuclear and mitochondrial genomes. Boro-Taichung (BT)-type CMS rice was obtained by substituting the nuclear genome of *Oryza sativa* subsp. *indica* cultivar Chinsurah Boro II with that of *Oryza sativa* subsp. *japonica* cultivar Taichung 65. In BT-type CMS rice, the mitochondrial gene *orf79* is associated with male sterility. A complete sequence of the Boro-type mitochondrial genome responsible for BT-type CMS has not been determined to date. Here, we used pyrosequencing to construct the Boro-type mitochondrial genome. The contiguous sequences were assembled into five circular DNA molecules, four of which could be connected into a single circle. The two resulting subgenomic circles were unable to form a reliable master circle, as recombination between them was scarcely detected. We also found an unequal abundance of DNA molecules for the two loci of *atp6*. These results indicate the presence of multi-partite DNA molecules in the Boro-type mitochondrial genome. Expression patterns were investigated for Boro-type mitochondria-specific *orf*s, which were not found in the mitochondria from the standard *japonica* cultivar Nipponbare. Restorer of fertility 1 (RF1)-dependent RNA processing has been observed in *orf79*-containing RNA but was not detected in other Boro-type mitochondria-specific *orf*s, supporting the conclusion that *orf79* is a unique CMS-associated gene in Boro-type mitochondria.

## Introduction

Plant mitochondria have large genomes with many repeat sequences and complex secondary structures. Because of its sequence complexity, the plant mitochondrial genome can create new sequences and new open reading frames (*orf*s) via homologous recombination. Expression of such newly formed *orf*s in mitochondria sometimes leads to cytoplasmic male sterility (CMS). Thus, such *orf*s are known as CMS-associated genes. A CMS-associated gene, in general, is composed of a part of a house-keeping mitochondrial protein-coding gene and a sequence from unknown origin, and encodes a peptide with a trans-membrane domain. In land plants, more than 150 species with CMS have been found in nature [1]. In plant breeding programs, CMS lines can be artificially obtained by successive backcrossing to substitute the nucleus of a female parent with that of a male recurrent parent. A female cytoplasm donor cultivar itself is fertile when it possesses *Restorer of fertility* (*Rf)* genes in its nuclear genome, which suppress the expression of CMS-associated genes. Loss of *Rf* genes by nuclear substitution enables the expression of CMS-associated genes and results in male sterility. Thus, this phenotype is most likely caused by incompatibility between the mitochondrial and nuclear genomes. The cloning of *Rf* genes thus far has revealed that most are involved in the regulation of transcripts of CMS-associated genes [2]. For example, the *Rf* genes of petunia CMS and radish CMS encode an RNA-binding protein known as pentatricopeptide repeat protein, which was found to bind the designated RNA of a CMS-associated gene and to post-transcriptionally suppressed the accumulation of a CMS-causing protein [3, 4, 5].

CMS is a useful phenotype in the breeding of F_1_ hybrid varieties, which provide 15%–20% higher yields than conventional inbred lines [6]. Employment of CMS eliminates the needs for hand emasculation to prevent the contamination of self-pollinated seeds. In rice, most of commercial F_1_ hybrid seeds are obtained based on a three-line system consisting of CMS, maintainer and restorer lines. The wild abortive (WA)-type CMS, which consists of the cytoplasm of *Oryza rufipogon* and the nuclear genome of *O*. *sativa* subsp. *indica*, has been most widely used for F_1_ hybrid rice production, and WA-type CMS-based hybrid rice has been planted on 6,000 kha in China [6]. The Boro-Taichung 65 (BT)-type CMS, which was obtained by combining the cytoplasm of *O*. *sativa* subsp. *indica* cultivar Chinsurah boro II and the nuclear genome of *O*. *sativa* subsp. *japonica* cultivar Taichung 65, has been also utilized for F_1_ hybrid rice, which has been planted on 130 kha in China [6]. It is also commercially utilized in Japan [7].

Because of its agronomic importance, many efforts have been made to identify the CMS-associated genes in mitochondria. Historically, the first approach in the identification of CMS-associated genes was through a comparison of genes and gene expression patterns between CMS and non-CMS mitochondria. The most conventional method for identifying CMS-associated genes relies on the use of northern blot screening to compare the transcript profiles of CMS and fertility-restored lines. Candidate genes are identified through detection of modified expression patterns in the presence of *Rf* genes (reviewed in [8]). Such examination of mitochondrial genes and gene expression has identified two CMS-associated genes in rice; *orf79* and its sequence variants in BT-type CMS [9–12], Lead rice (LD)-type CMS [13] and Honglian (HL)-type CMS [14], and *orf352* in WA-type CMS [15].

The most well-characterized CMS-associated gene in rice is *orf79* in BT-type CMS [9–13, 16]. It encodes a 79 amino acid peptide with a transmembrane domain and carries a chimeric structure composed of part of a gene for mitochondrial cytochrome oxidase subunit I and a sequence of unknown origin [10]. It has been reported that Boro-type mitochondria contain two duplicated loci of the *atp6* gene, which encodes a subunit 6 of the ATPase synthase. One locus is completely identical to the Nipponbare *atp6* locus (N-*atp6*). Another locus of *atp6* (B-*atp6*) can be distinguished from N-*atp6* from the differences in the sequence at its 3′ untranslated region. The *orf79* gene exists 208-nt downstream of B-*atp6*. In Boro-type mitochondria, the B-*atp6* is co-transcribed with *orf79* as a 2.0-kb RNA, whereas a 1.5-kb transcript containing *atp6* and a 0.45-kb transcript containing *orf79* are generated through RNA processing under the presence of a *restorer of fertility* gene, *Rf1*, which encodes a pentatricopeptide repeat protein [12, 16].

Recently, next-generation sequencing has become a popular method to uncover CMS-associated genes. Whole mitochondrial genome sequences have been compared to those of standard cultivars such as Nipponbare to screen for new *orf*s that were absent in the reference genome [17–20]. CMS-associated gene candidates have been selected based on whether they carry chimeric structures with known mitochondrial genes or encode proteins with a transmembrane domain, and whether or not they exhibit different expression patterns in the presence or absence of *Rf* genes. Whole mitochondrial genome sequences have been reported for Nipponbare [21], PA64S [22], *indica* cultivars 93–11 [22], IR6888B [17], and Hassawai [23], and several CMS lines such as LD-type CMS derived from *Oryza sativa*, WA-type CMS, Chinese wild rice (CW)-type CMS, RT98-type CMS, and RT102-type CMS derived from *Oryza rufipogon* [17–20]. Whole genome sequences of Boro-type mitochondria, however, have not been reported to date despite its economic importance in the production of F_1_ hybrid seeds.

In this study, we determined the Boro-type mitochondrial genome sequence responsible for BT-type CMS using a Roche 454 GS pyrosequencing system, a next-generation sequencing platform. Sequence assembly indicated that the mitochondrial genome has a flexible multi-partite structure. Northern blot analysis was performed on Boro-type mitochondria-specific *orf*s to explore the possibility of RF1-regulated RNA processing in *orf*s other than *orf79*.

## Results and Discussion

### Genome structure, size, genes and *orf*s of Boro-type mitochondria

**Fig 1:**
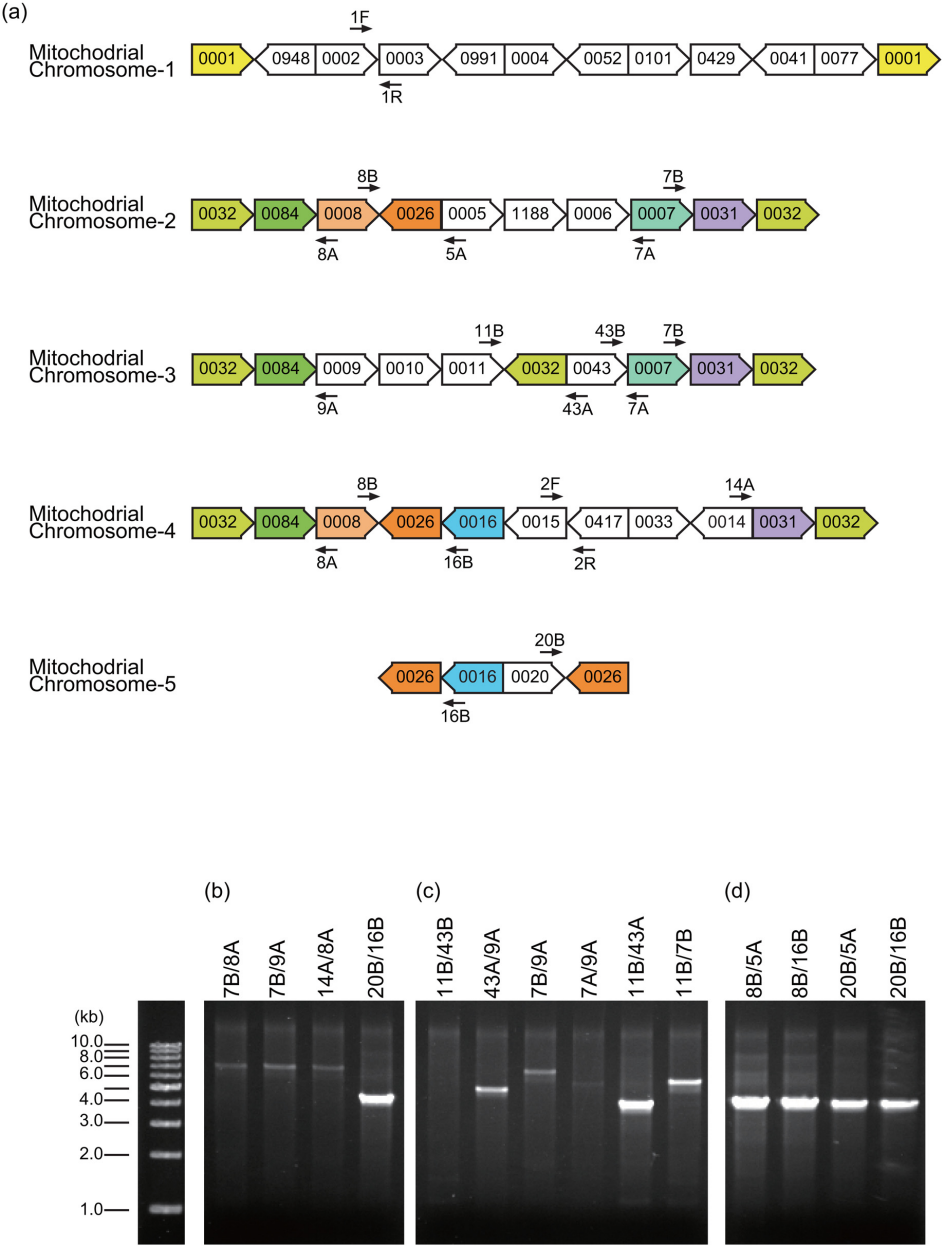
Boro-type mitochondria sequences are assembled into five circular chromosomes. (a) Compositions of contigs in five assembled mitochondrial chromosomes (MC). The arrow box indicates a contig and its direction. Arrows represent primers used for confirmation of contig connections. The same contigs are filled with the same colors. (b) Each of the five circular chromosomes was validated by PCR analysis using the primer sets indicated. (c) The inverse order of the contigs in MC-3 was detected by PCR analysis. (d) Integration of MC-5 into MC-2 was validated by PCR analysis.

**Fig 2:**
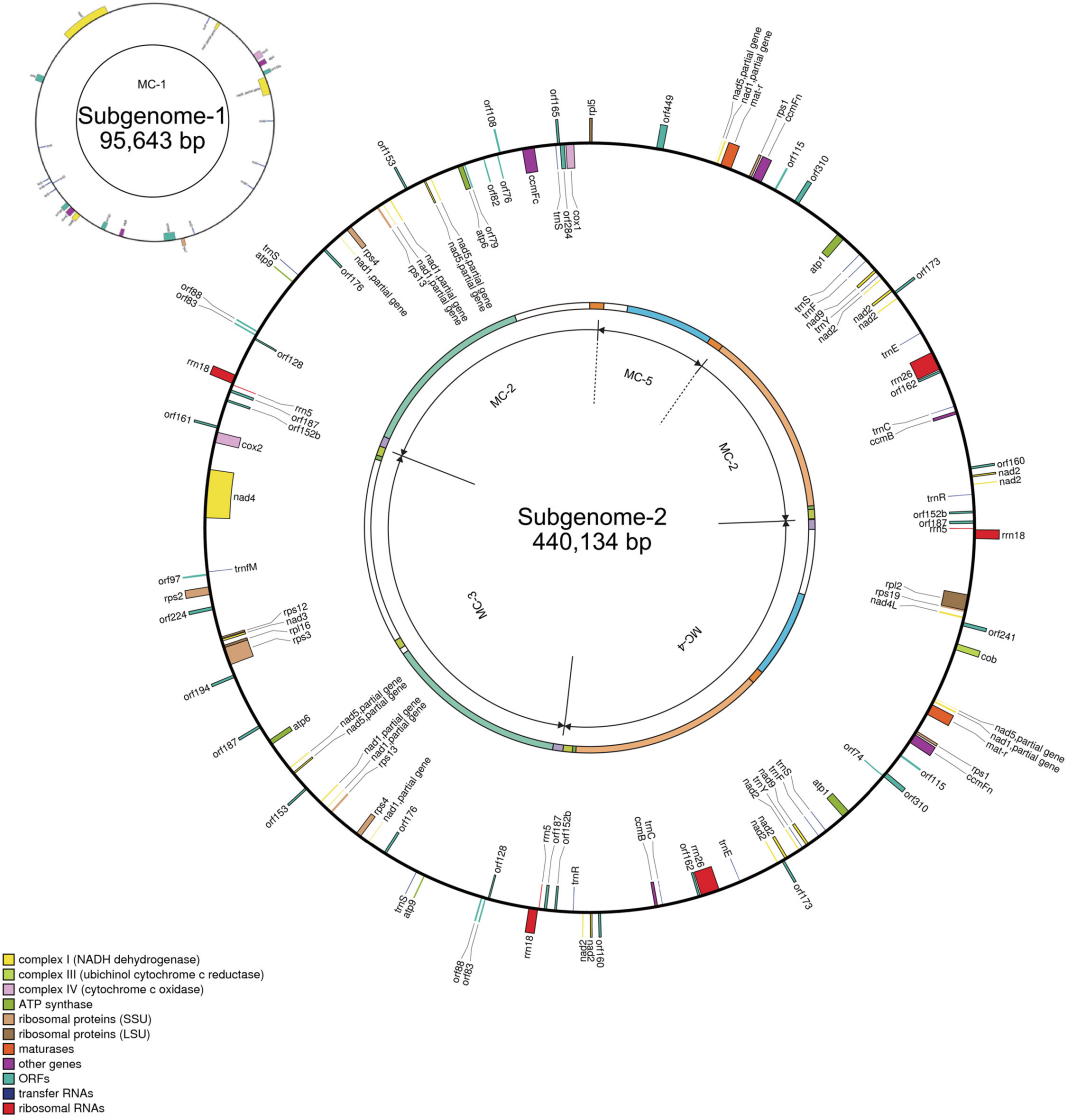
Graphical genome maps of subgenome-1 and subgenome-2 with all of the known genes and Boro-type mitochondria-specific *orf*s. The outer circle indicates the graphical genome maps generated by using OGdraw. Colors in the inner circle correspond to those of the contigs listed in Fig 1.

**Fig 3:**
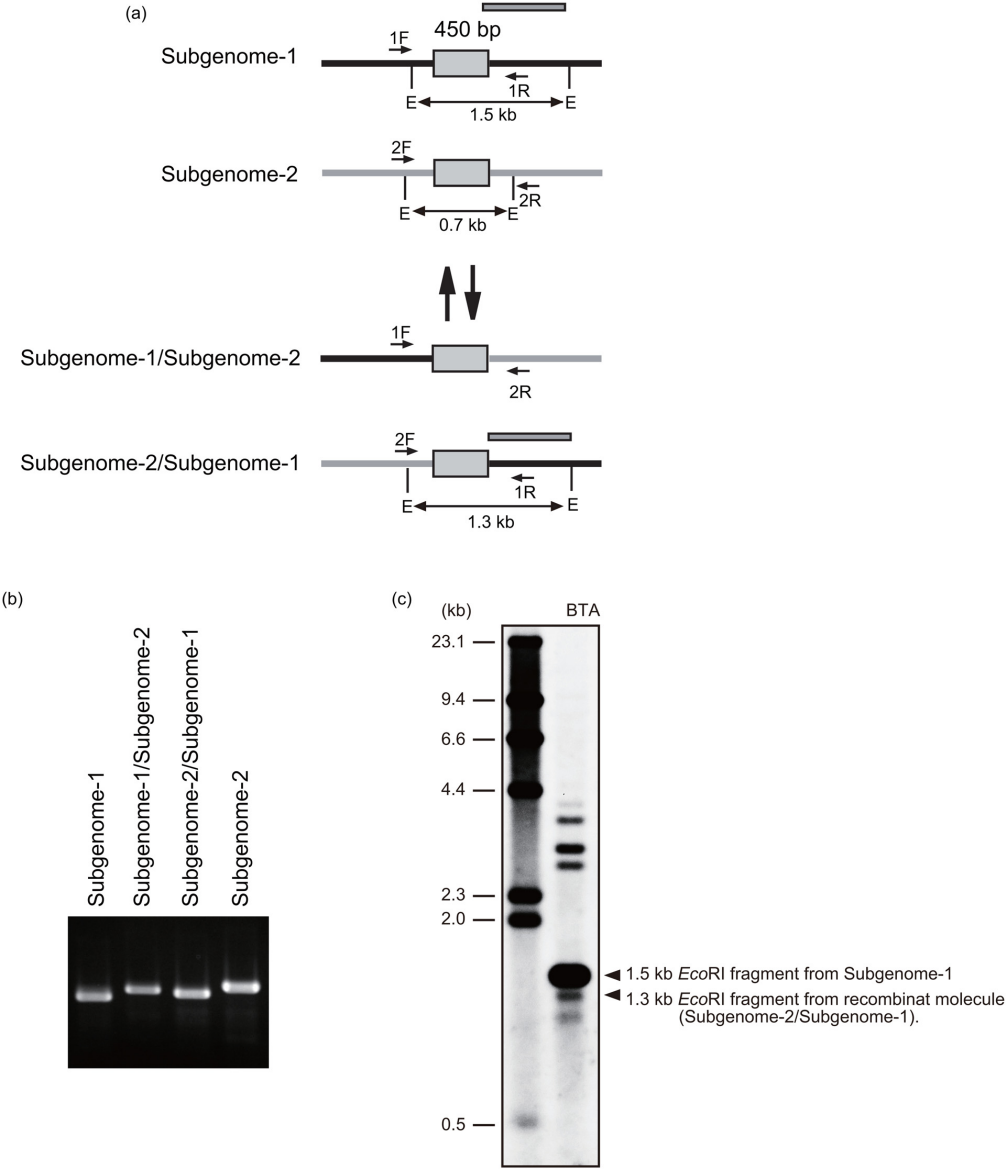
Recombination between subgenome-1 and subgenome-2 via the 450-bp identical sequence is possible but rare. (a) Schematic model of recombination between subgenome-1 and subgenome-2. Arrows indicate primers used for detection of recombination. *Eco*RI sites are indicated as E. (b) Recombinant fragments were detected by PCR. (c) Southern blot analysis detected the recombination molecule between subgenome-1 and subgenome-2; however, the frequency of recombination was lower than that of the non-recombinant subgenome-1 molecule.

Using contiguous sequences (contigs) and bridge sequences obtained from pyrosequencing, we assembled the Boro-type mitochondria genome sequences into five circular DNA molecules (Fig 1a and S1 Table). We named them Mitochondrial Chromosome 1 to 5 (MC-1, MC-2, MC-3, MC-4, and MC-5; Fig 1a). The circular molecules of MC-2 to MC-5 were validated by PCR analysis using sequence-specific primer sets 7B/8A, 7B/9A, 14A/8A and 20B/16B, respectively (Fig 1b). An inverse order of the contiguous contigs flanked by the inverted repeats of contig0032 in MC-3 (contigs 0032-0031-0007-0043-0032) was also possible; however, we adopted the order presented in Fig 1a because this order yielded more intense PCR amplification using primer set 7B/9A compared to that using 7A/9A (Fig 1c). Large, perfect repeats were shared between each mitochondrial chromosome (Fig 1a). MC-5 could be integrated into both MC-2 and MC-4 by recombination at contig0026, a high-read-depth contig. Integration of MC-5 into MC-2 was detected by PCR analysis using primer sets 20B/5A (Fig 1d). Strong band intensity of the amplicon using primer set 20B/5A supported the presence of such an integrated molecule. MC-2 integrated with MC-5, MC-3 and MC-4 can form a large single circular molecule where the components are connected to each other via the high-read-depth contig, contig0032. Amplification of approximately 7.3-kb bands using primer pairs 7B/8A, 7B/9A and 14A/8A (Fig 1b) supported the possibility of the presence of a connected circular molecule of MC-2, MC-3 and MC-4, as well as the possibility of the presence of each circular chromosome. Finally, the Boro-type mitochondrial genome was assembled into two large circular subgenomes named subgenome-1 and subgenome-2 (Fig 2). Subgenome-1 consists of MC-1 and was approximately 95 kb in length. Subgenome-2 was composed of MC-2, MC-3, MC-4, and MC-5 and was approximately 440 kb long (Figs 1 and 2). No common contigs were shared between subgenome-1 and subgenome-2. However, a 450-bp identical sequence was found at the junction of contig0002 and contig0003 in subgenome-1 and at the contig0417 side of contig0015 in subgenome-2 (S1 Fig). This suggests that subgenome-1 and subgenome-2 could form a master circle structure if recombination occurs at these identical sequences (Fig 3a). To confirm this hypothesis, we performed PCR analysis using primer sets targeting this identical sequence to detect a possible recombination event (Figs 1a, 3a and 3b). To obtain a direct evidence for this recombination, Southern blot analysis was also performed using a probe targeting the sequence just adjacent to the identical region in subgenome-1 (Fig 3a). After *Eco*RI digestion, 1.5-kb major and 1.3-kb minor bands were detected (Fig 3c), which could have originated from subgenome-1 and the recombinant DNA molecule, respectively. This result indicates that both forms of the genome exist *in vivo*; however, the subdivided form of subgenome-1 is likely to be the more stable form *in vivo*. DNA fragments with sizes of 2.3 kb to 4.4 kb were also detected (Fig 3c), suggesting that another order of contigs could exist or that sub-stoichiometric shifting had occurred. A similar phenomenon has been reported in cucumber mitochondria, in which the large circular chromosomes favor subdivision by homologous recombination via direct repeat sequences [24].

B-*atp6* and *orf79* were found in contig0007 and contig0006 of MC-2 with read depths of 97.8 and 112.2, respectively (S1 Table). In contrast, the depth of contig0043 that corresponded to a sequence 49-bp downstream of N-*atp6* was as low as 12.1 (S1 Table, S2 Fig). The existence of an overlapping sequence in contig0007 and contig0043 was supported by the reported sequences of the N-*atp6* locus (DDBJ accession number S59890.1), which was also present in other mitochondrial genomes in S2 Table, except that of LD-CMS. These results suggest that the abundance of molecules including N-*atp6* is approximately 10% of that for B-*atp6*. A lower abundance of N-*atp6* was supported by the weaker signal intensity of N-*atp6* compared to that of B-*atp6* in the Southern blot analysis of an *Eco*RI-digested mitochondrial genome probed with *atp6* (S2 Fig), as previously reported [9, 10, 13]. These results suggest the presence of multi-partite molecules and the possibility that a fragment flanked by an inverted repeat of contig0032 in MC-3 (contigs 0032-0043-0007-0031-0032) might exist as a linear molecule.

It must be noted that a 3,041-bp-long sequence completely identical to the one found 78 bp upstream of the *atp6* gene to the end of contig0043 (S2 Fig) was also found in the chromosome 1 of Nipponbare (position 33,552,397..33,549,357 in The Rice Annotation Project Database). In ancestral species, the N-*atp6* sequence may have been transferred from mitochondria to the nucleus and integrated into the nuclear genome. In Boro-type mitochondria, the N-*atp6* sequence may be evolutionarily in the process of disappearing from the mitochondrial genome, starting from the time at which the B-*atp6-orf79* locus was created. The N-*atp6* locus is lost in LD-type mitochondria, which only have a B-*atp6-orf79* locus [18]. Further studies are required to define how N-*atp6* and B-*atp6* are maintained in Boro-type mitochondria.

We also obtained five plasmid-like small molecules (S1 Table) that have been previously reported as B1 (2,135 bp; DDBJ accession D00293), B2 (1,548 bp; DDBJ accession D00564), B3 (1,514 bp; DDBJ accession X16154) and B4 (969 bp; DDBJ accession X07904). The GC content of each plasmid-like molecule was 44.2%, 42.9%, 42.1%, and 49.8%, respectively, which was almost identical to that of subgenome-1 (44.0%) and subgenome-2 (43.9%). These values are almost comparable to the GC content of 43.8% in the mitochondrial genome of Nipponbare [22], indicating that the Boro-type mitochondrial genome has no distinct feature with regard to GC content.

Almost all of the mitochondrial genes reported by Notsu et al. [21] exist in the Boro-type mitochondrial genome, although exceptions include *orf288* and pseudo-*rps14*. *orf288* in Nipponbare mitochondria, changed into *orf310* because of a nucleotide insertion. pseudo-*rps14* lost six nucleotides in Boro-type mitochondria. The exon and intron structures of each gene have been conserved. The *nad1* and *nad5* genes are composed of five exons; each exon is transcribed separately and is processed via trans-splicing to form the functional message. Interestingly, in Boro-type mitochondria, exon 1 of *nad1* and *exon 1* and *exon 2* of *nad5* are in subgenome-1. The residual exons (*exon 2* to *exon 5* of *nad1* and *exon 3* to *exon 5* of *nad5*) are in subgenome-2. These findings suggest that when the mitochondrial genome is transmitted to the next generation or daughter cells, both of the two subgenomes should be transmitted equally to maintain the expression of these genes.

### Some Boro-type *orf*s are not found in the Nipponbare mitochondrial genome

Once constructed, the Boro-type mitochondrial genome was then screened for the presence of *orf*s that encoded proteins longer than 70 amino acids because the CMS- associated gene *orf79* encodes a 79-amino-acid protein. More than 600 *orf*s were predicted in two subgenomes. A BLAST search identified 17 *orf*s that were not found in Nipponbare mitochondria (S2 Table). We selected all of these *orf*s for analysis as Boro-type mitochondria-specific *orf*s. The *orf*s were named based on the length of the proteins they encoded. Ten of these *orf*s were conserved as full-length coding regions in other types of CMS mitochondria (S2 Table). *orf76*, *orf79*, *orf82*, and *orf310* were only found in LD-type mitochondria [20]. We found *orf115* in RT98-, LD-, ZS97-, and WA-type CMS mitochondria [13, 20, 21]. *orf83*, *orf88*, and *orf128* were detected in RT98-, RT102-, LD-, CW-, and WA-type CMS mitochondria [15]. The partial fragments of *orf74*, *orf97*, *orf108*, *orf310*, and *orf449* completely matched those found in the Nipponbare mitochondrial genome, however. Additionally, the plasmid-like small molecules carried *orf80*_B1, *orf95*_B1, *orf138*_B2, *orf73*_B3 and *orf83*_B4, all of which were found in the plasmid-like small molecules in LD-type and CW-type CMS rice [18]. These results indicate that the *orf*s characteristic for Boro-type mitochondria are restricted to a set of *orf79*, *orf82*, *orf76* and *orf108*, which are adjacent to each other between the B-*atp6* and *ccmFc* loci in conting0006 (Fig 2).

Chimeric features of gene structures were found in *orf449*, *orf310*, *orf97*, and *orf79* (S2 Table). *orf449* contains a partial sequence of the *cob* gene that encodes cytochrome b. This *orf449* has already been deposited in DNA databases as a gene for pseudo apocytochrome b (DDBJ accession X53711). *orf310* contains a partial sequence identical to *orf288* and is predicted to encode a hypothetical protein. *orf97* contains a partial sequence almost identical to that of *orf25* (*atp4*), which encodes a subunit of ATP synthase.

### Expression analysis of *orf*s specific to Boro-type mitochondria

**Fig 4:**
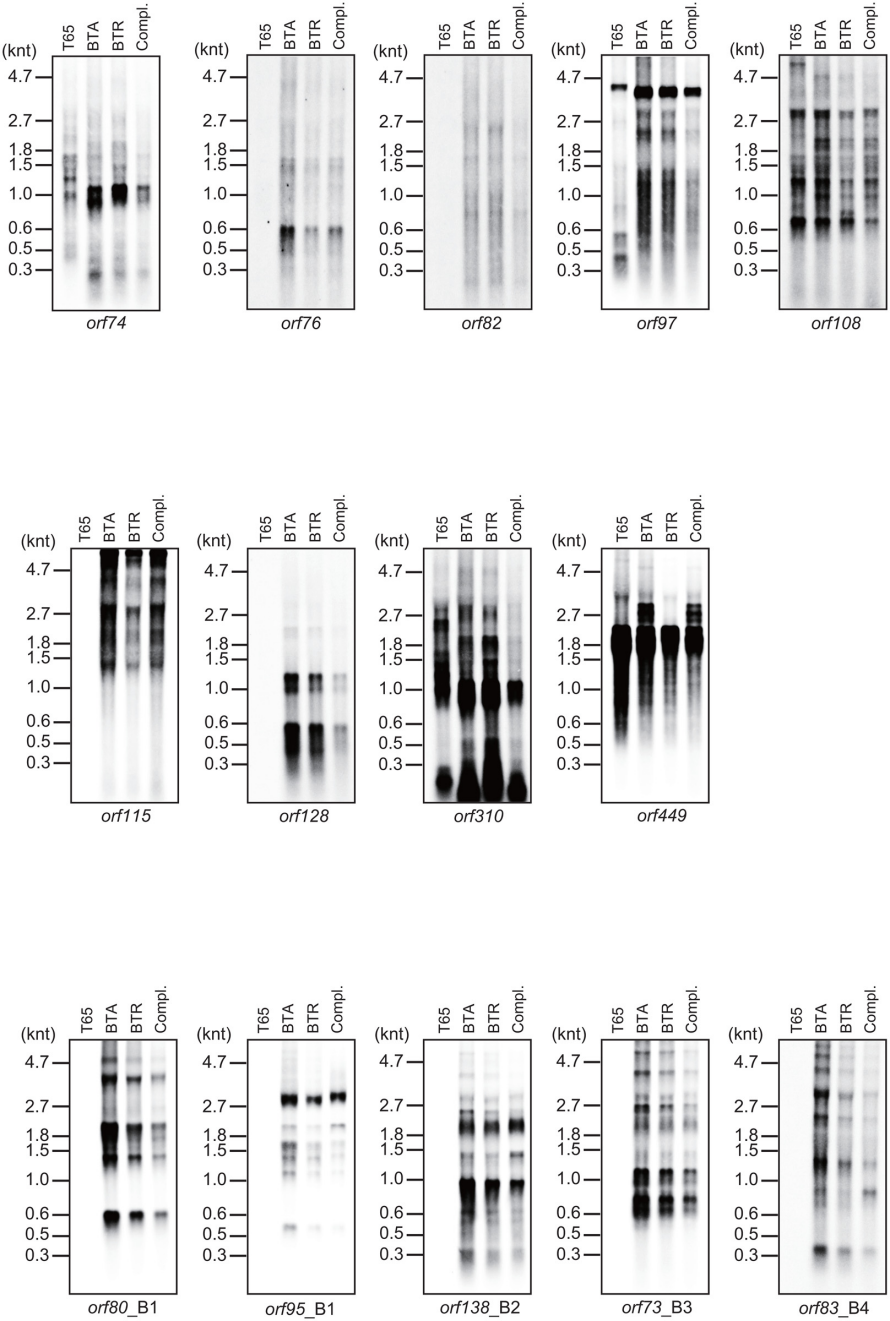
Expression patterns of *orf*s specific to Boro-type mitochondria were not altered in the presence or absence of the restorer of fertility gene, *Rf1*. Northern blot analysis was performed using a BT-type CMS rice (BTA), a fertility restorer with *Rf1* (BTR), a transgenic BTA complemented with *Rf1* (Compl), and a maintainer line (T65).

To determine whether or not the expression pattern of these *orf*s is dependent on the presence of the fertility restorer gene, *Rf1*, we performed northern blot analyses on the 16 *orf*s (*orf74*, *orf76*, *orf82*, *orf83*, *orf88 orf97*, *orf108*, *orf115*, *orf128*, *orf310*, *orf449*, *orf80*_B1, *orf95*_B1, *orf138*_B2, *orf73*_B3, and *orf83*_B4) using the BT-type CMS rice (BTA), fertility restorer rice (BTR) with *Rf1*, a transgenic BTA line transformed with *Rf1* (complemented line) and a maintainer line (T65) (Fig 4). Transcripts of the 14 *orf*s were detected, except for *orf83* and *orf88* in BTA, BTR, and the complemented line carrying Boro-type mitochondria. We expected that CMS-associated genes would show different transcript profiles for the CMS BTA line compared to those of the fertility-restored BTR line and the complemented line, depending on the absence or presence of *Rf1*. However, we did not detect an *Rf1*-dependent alteration of expression patterns. These results suggest that the *orf*s shown in Fig 4 are not likely to be CMS-associated genes, thus supporting the previous reports that *orf79* is the unique CMS-associated gene in Boro-type mitochondria [9, 10].

In our previous study, we reported that the recombinant RF1 protein bound to a 370-nt long RNA probe termed BD4, whose sequence corresponded to the intergenic region between *atp6* and *orf79* RNA [16]. Thus, there was a possible RF1 target sequence in this 370-nt sequence. A similarity search comparing the BD4 sequence with the Boro-type mitochondria sequence revealed that a 67-bp-long sequence in the BD4 region was identical to the 5’ region of *orf97* that was 16 bp upstream from the start codon (S3 Fig). This implied that the transcript pattern of *orf97* could be altered by the presence of *Rf1*. However, we did not detect any differences when northern blot analysis was performed using an *orf97* probe (Fig 4). We have concluded that the 67-bp region does not contain a binding sequence for RF1. This indicates that *orf97* is not a CMS-associated gene of BT-type CMS rice, further supporting the previous finding that *orf79* is a unique CMS-associated gene post-transcriptionally affected by RF1 [9, 10].

## Conclusion

Pyrosequencing of the Boro-type mitochondrial genome revealed the presence of multipartite structures. The abundance of N-*atp6* molecules is quite low compared to that of B-*atp6* molecules, which suggests these structures are a unique feature of the Boro-type mitochondrial genome. We conclude that *orf79* is a unique CMS-associated gene in BT-type CMS. The whole-genome sequencing data of Boro-type mitochondria will provide useful information for comparative studies of CMS-causing genetic factors in rice.

## Materials and Methods

### Plant materials

The BT-type CMS rice, BTA, was made from the cytoplasm of the *indica* cultivar ‘Chinsurah Boro II’ with the nuclear background of the *japonica* cultivar ‘Taichung 65’. BTR is an isogenic restorer line, except for the *Rf1* locus, and contains the *Rf1* allele [16].

### DNA extraction and Southern blot analysis

Total DNA was extracted from a leaf blade using the DNeasy Plant Mini Kit (Qiagen, https://www.qiagen.com/). Mitochondrial isolation was performed according to the description provided in a previous study [25]. Mitochondrial DNA was extracted using a DNeasy Plant Mini Kit (Qiagen). To investigate recombination events between subgenome-1 and subgenome-2, 1 μg of total DNA was digested with *Eco*RI. Gene-specific DNA probes were produced using the PCR DIG Labeling Mix (Roche) by amplification of each gene using the following primer sets: sub1_rec_F1 (5′- CCATGGAATGACGGAATTCC-3′) / sub1_rec_R1 (5′- AGGATAAGGGGGAGTAGTAG-3′). Hybridization, washing, and detection were performed according to the manufacturer’s instructions for the DIG DNA labeling and the detection kit (Roche). Southern blot analysis for the detection of *atp6* was performed using 300 ng of mitochondrial DNA by a method described previously [25].

### Sequence assembly

Pyrosequencing was performed using a GS-FLX system with 8-kb paired-end adoptees (Roche, https://roche-biochem.jp/) at TaKaRa Bio (http://www.takara-bio.co.jp/). This analysis resulted in 67,784,793 bases for the Boro-type mitochondrial genome and the sequence were assembled into 1,412 contigs and 26 scaffolds, using GS De Novo Assembler version 2.3 (Roche). After eliminating nuclear genomic contamination, we adopted a reference sequence-based assembly using the Nipponbare mitochondrial genome (DDBJ accession No. BA000029; [21]) as a reference genome, and as described previously [17]. The contigs were then manually assembled based on the scaffolds and the presence of bridge contigs, and validation was performed by PCR analysis in the same manner as described previously [18]. Ambiguous sequences of protein-coding regions that were distinct from those of Nipponbare, were confirmed by direct sequencing of the PCR products using a CEQ8000 genetic analyzer (Beckman Coulter, http://www.beckmancoulter.co.jp/). The PCR primers used for the confirmation of contig linkages are listed in S3 Table. The PCR reaction was performed using the leaf-derived total genomic DNA as a template and Ex-Taq polymerase (TaKaRa Bio). All of the contiguous contigs, together with each read depth, are listed in S1 Table. Two hypothetical master circles were developed using a ‘parsimonious’ method, such that each contig appeared at least once when constructing the smallest genome. Other configurations can be considered from rearrangements at sites identical to those of the high-read-depth contigs. The graphical genome map was generated using OGDraw software (http://ogdraw.mpimp-golm.mpg.de/; [26]).

### Specific *orf*s in Boro-type mitochondrial genome

The potential *orf*s were identified using Artemis software [27], which uses a threshold to identify ORFs, as previously used in an analysis of the WA-type CMS mitochondrial genome [17]. In the present study, we used 70 amino acids as the threshold, because the shorter mitochondrial peptides that cause CMS in rice such as *orf79* contain 79 amino acids. Common *orf*s with >99% identity to those in Nipponbare were identified using Sequencher software (Gene Codes Corporation, https://www.genecodes.com/) and were subsequently discarded. The TMHMM sever v3.0 (http://www.cbs.dtu.ck/services/TMHMM/; [28]) was used to predict transmembrane domains in proteins encoded by *orf*s.

### RNA extraction and northern blot analysis

Secondary calli were transferred into fresh medium every 2 weeks, and 4-day-cultured calli were subjected to total and mitochondrial RNA extraction. Total RNA was extracted using an RNeasy Plant Mini Kit (Qiagen). DNA was eliminated using RNase-free DNase I (TaKaRa Bio). RNA (1 μg) was treated with Superscript III reverse transcriptase (Invitrogen, http://www.thermofisher.co.jp/) to produce complementary DNA. The mitochondrial isolation was performed according to a previous description [29], and RNA was extracted by RNAiso-Plus (TaKaRa Bio). Northern blot analysis was performed as previously described [12]. Gene-specific DNA probes were produced with PCR DIG Labeling Mix (Roche) by amplification of each *orf* using the primer sets listed in S3 Table. Signals were detected and measured using ImageQuant LAS 4000 mini (GE Healthcare Life Sciences, http://www.gelifesciences.co.jp/).

## Supporting Information

S1 Fig An identical 450-bp-long sequence shared between subgenome-1 and subgenome-2. Sequence alignment of the identical genomic region between subgenome-1 and subgenome-2.
(EPS)

S2 Fig Comparison of N-*atp6* and B-*atp6-orf79* indicates a lower abundance of N-*atp6* molecules in the Boro-type mitochondrial genome. (a) Schematic representation of N-*atp6* and B-*atp6*-*orf79* loci in contig0006-contig0007 and contig00043-contig0007, respectively. (b) Southern blot analysis of the *Eco*RI-digested mitochondrial genome probed with *atp6* and *coxI*. The *coxI* probe was used as an internal control.
(EPS)

S3 Fig A sequence included in the intergenic region of *atp6-orf79* RNA was found in the 5’ upstream region of *orf97*. BD4 is a 370-nt-long RNA probe that binds the RF1 protein. A 67-bp-long sequence in the BD4 region is identical to a 16-bp upstream region of *orf97*.
(EPS)

S1 Table Compositions of mitochondrial chromosomes (MC-1 to MC-5) in subgenome-1 and subgenome-2, as well as plasmid-like molecules. (XLSX)

S2 Table Characteristics of *orf*s specific to Boro-type mitochondria. (XLSX)

S3 Table Primers used for validation of the contig linkage. (XLSX)

S4 Table Primer sets used for synthesizing probes in Southern and northern blot analyses of *orf*s specific to Boro-type mitochondria. (XLSX)
